# Sustainability Characterization for Additive Manufacturing

**DOI:** 10.6028/jres.119.016

**Published:** 2014-09-22

**Authors:** Mahesh Mani, Kevin W Lyons, SK Gupta

**Affiliations:** 1 National Institute of Standards and Technology, Gaithersburg, MD 20899; 2 University of Maryland, College Park, MD 20742

**Keywords:** additive manufacturing, characterization, performance metrics, standardization, sustainability

## Abstract

Additive manufacturing (AM) has the potential to create geometrically complex parts that require a high degree of customization, using less material and producing less waste. Recent studies have shown that AM can be an economically viable option for use by the industry, yet there are some inherent challenges associated with AM for wider acceptance. The lack of standards in AM impedes its use for parts production since industries primarily depend on established standards in processes and material selection to ensure the consistency and quality. Inability to compare AM performance against traditional manufacturing methods can be a barrier for implementing AM processes. AM process sustainability has become a driver due to growing environmental concerns for manufacturing. This has reinforced the importance to understand and characterize AM processes for sustainability. Process characterization for sustainability will help close the gaps for comparing AM performance to traditional manufacturing methods. Based on a literature review, this paper first examines the potential environmental impacts of AM. A methodology for sustainability characterization of AM is then proposed to serve as a resource for the community to benchmark AM processes for sustainability. Next, research perspectives are discussed along with relevant standardization efforts.

## 1. Introduction

**Fig. 1:**
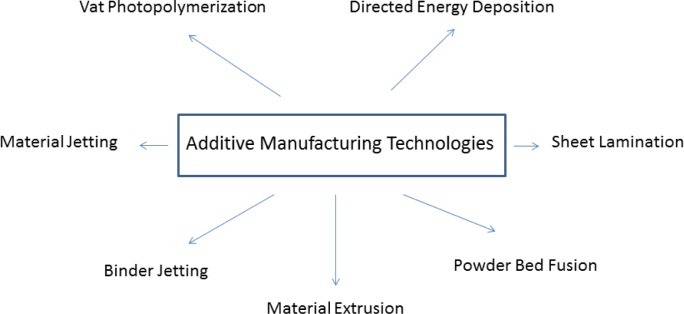
AM process technology categories as per ASTM F42.

Additive Manufacturing is increasingly used in the development of new products, spanning conceptual design, functional parts, and tooling. Additive Manufacturing (AM) as it is now called according to ASTM International [1] is also referred to as rapid prototyping, additive fabrication, freeform fabrication, 3D printing, and rapid manufacturing; and uses advanced technologies to fabricate parts by joining and building up material layer-by-layer. AM is an emerging technology and has shown promise in applications as diverse as biomedical implants to aerospace and automotive industry [2–9]. The ability of AM to build parts directly from a digital representation makes it an excellent alternative compared to traditional manufacturing like machining, injection molding, and die-casting for rapidly making highly customized parts. Note that while AM is inherently suited for making products with complex geometry in relatively small volumes, it has shown that it can conveniently accelerate mass production by making tools and dies used in large volume manufacturing. It can also accelerate the production of selected parts by combining multiple parts into one. The variety of raw materials used in AM currently includes metallic, plastic, ceramic, or composite materials in different forms, such as powders, wires, or liquid. Due to the differences in AM technologies and processes, functional and geometrical properties of manufactured parts can vary dramatically. Planning decisions to select the appropriate AM process and material for specific application requirements are rather involved [10]. Several researchers have well documented the opportunities of AM processes and techniques, their applications in diverse industry sectors, and the influence of AM in production systems to enable rapid manufacturing and mass production [11–13]. However, research is still required to fully realize the potential of AM processes to overcome the barriers of cost for materials property data, qualification, and certification, particularly for critical components (aerospace parts or automotive parts). AM processes that have been previously categorized by different researchers [3, 14–16] and have now been standardized by the ASTM International Committee F42 on AM Technologies into the seven classes as shown in Fig. 1 [1]. According to [17], “the expected long-term impact is in highly customized manufacturing, where AM can be more cost-effective than traditional methods. According to an industry report by Wohlers Associates, by 2015 the sale of AM products and services could reach $3.7 billion worldwide, and by 2019, exceed $6.5 billion.”

## 2. Additive and Sustainable Manufacturing

Sustainable manufacturing is defined as the creation of manufactured products that use processes that minimize negative environmental impacts, conserve energy and natural resources, are safe for employees, communities, and consumers, and are economically sound [18]. When assessing sustainability of AM process or any manufacturing process, one must consider the entire life cycle to arrive at the sustainability impact. The actual manufacturing process is only one of the many environmental impacts associated with the product life cycle.

There is not much hard data yet on how AM products compare to traditionally manufactured products in terms of energy use, supply chain, pollution, and other potential environmental impacts over their lifetime [19].

The manufacturing sector accounts for approximately one-third of U.S. energy consumption [20, 21]. Reducing energy consumption and associated energy costs through increased energy efficiency measures helps strengthen the economic vitality of U.S. manufacturers while also helping to protect our environment. Energy efficiency, as well as the cost and availability of energy, consequently have a substantial impact on the competitiveness and economic health of U.S. manufacturers [18]. Table 1 presents some key AM advantages relevant to sustainable manufacturing.

## 3. Literature Review on Environmental Impacts of AM

Bourell et al. [22] presented a historical perspective along with the most recent developments in the field of the AM technology. They further reported on a U.S. national roadmap workshop to define research needs and possibilities for AM in the next 10–15 years. This research documented several AM research recommendations, including some in energy and sustainability applications. Specific research recommendations included: design energy system components to take advantage of AM capabilities; pursue maintenance, repair, and overhaul (MRO) as a potential AM application; develop equitable indicators for measuring sustainability in AM processes and products; and identify sustainable engineering materials for AM processes.

More recently, the Roadmap Workshop on Measurement Science for Metal-Based Additive Manufacturing sponsored by the National Institute of Standards and Technology (NIST) explored the challenges that impede AM widespread adoption, particularly in the areas of measurement science and standardization [23]. With reference to sustainability, the roadmap highlighted that AM could greatly reduce the waste in manufacturing, reducing the energy used in production of raw materials and in the processing steps. By using AM for repair and remanufacturing, not only will the material waste and amount of landfill be reduced, but also energy and matter consumption during manufacture will be reduced because existing components are utilized. The roadmap also identified a lack of measurement standard for total energy input and losses for AM.

Baumers et al. [24] presented a comparative assessment of the electricity consumptions of two major polymeric Laser Sintering platforms^1^: the Sinterstation HiQ + HS from 3D Systems and the EOSINT P 390 from EOS GmbH. The energy inputs for building two prosthetic parts were recorded during power-monitoring experiments conducted on both platforms. The paper presented AM energy usage as job-dependent, time-dependent, geometry-dependent, and Z-height-dependent energy consumption values. In another paper [25], the authors presented an overview of electrical consumption across several major AM technology variants, reporting specific energy consumption during the production of dedicated test parts.

Diegel et al. [26] presented how aspects of AM, from a sustainable design perspective, could become a useful tool to bring about the sustainable design of consumer products. The paper emphasized the relationship between design quality and sustainability. Some of the principal design considerations to keep in mind when designing for AM include: enclosed voids, surface finish, strength and flexibility, and machine and material cost.

Scott et al. [10] presented the state of the industry for AM and in particular the technical challenges and emerging research and development in the area. The technical challenges included material characterization, material development, process control, process understanding and modeling, machine qualification, machine modularity, and design tools and software. With reference to the environmental impact, the report highlighted the need to develop equitable metrics for measuring the environmental impacts and sustainability of AM processes.

Hao et al. [27] presented research activities on sustainable product design by optimizing internal lightweight structures; process efficiency improvement by optimizing AM process parameters; reduction of energy consumption by *in-situ* material reaction; and sustainable production of personalized chocolates.

Faulkner et al. [28] presented a methodology to prepare a sustainable value stream mapping which includes various metrics to evaluate not only the economic performance, but also the environmental and societal sustainability performance of a manufacturing line. Metrics were selected to assess process water consumption, raw material usage, energy consumption, potential hazards concerning the work environment, and the physical work done by the employees.

Ellis and Hadley [29] proposed that with a wider suite of metrics in remedial program decisions, more holistic and sustainable decisions are made. Reducing the inherent consumption of energy, raw materials, and other consumables is the most significant opportunity for implementing more sustainable remedial actions. The traditional remedial technology evaluation process does not assess greenhouse gas emissions, natural resource consumption, energy use, worker safety, and/or local and regional impacts.

Sustainability indicators and composite index are increasingly recognized as a useful tool for policy making and public communication in conveying information on country and corporate performance in fields such as environment, economy, society, or technological improvement. Singh et al. [30] proposed by visualizing phenomena and highlighting trends, sustainability indicators simplify, quantify, analyze, and communicate otherwise complex and complicated information.

Kinderyte [31] presented an approach to assess sustainability of printing enterprises. The approach consisted of qualitative and quantitative parts and a composite index. Qualitative and quantitative parts are aggregated into one improved sustainability index. The main areas identified for improvement in printing enterprises were: use of more ecological paper, paints, and other resources; reduction of volatile organic compounds and use of renewable energy.

Sreenivasan et al. [32] presented an overall energy assessment for selective laser sintering (SLS) of polymers using eco-indicators. They also presented the electrochemical deposition of porous SLS non-polymeric preforms with a goal to reduce energy consumption in SLS of non-polymeric materials.

Hiller and Lipson [33] demonstrated a freeform fabrication system that prints with fully reusable physical voxels and minimal recycling. This new paradigm of digital (discrete) matter enables any number of materials to be printed together in any configuration. The research presents opportunities for flexible desktop fabrication processes in which 3D multi-material objects are fully recyclable and re-usable with minimal infrastructure.

Nopparat and Kianian [34] investigated the benefits of accessing the AM technology through the result-oriented Product-Service Systems (PSS) approach in the scale model kit industry, thereby quantifying raw materials and energy consumption. The result shows that AM has higher efficiency in terms of raw material usage, however it has higher energy consumption in comparison to the more traditional manufacturing techniques.

Le Bourhis et al. [35] presented a new methodology where all flows consumed (material, fluids, electricity) are considered in the environmental impact assessment. The proposed method coupled a global view required in a sustainable approach and an accurate evaluation of flow consumption in the machine. The methodology developed was based on a predictive model of flow consumption defined from the manufacturing path and Computer Aided Design (CAD) model of the part that will be produced.

Diegel et al. [26] examine how aspects of AM, from a sustainable design perspective, could become a useful tool to bring about sustainable design of consumer products. As AM technologies evolve, and more new materials become available, and multiple material technologies are further developed, the field of product design has the potential to greatly change.

Bertling et al. [36] presented the sustainability aspects for two distinct development directions in Direct Digital Manufacturing: the replacement of industrially established processes by AM and the FabLab movement as an example of a paradigm shift in consumer-producer-relationship. In general, the paper suggested that in order to reduce the ecological footprint of consumption decisively, the whole system of producing and consuming needs to be innovated. The study also suggested that participation, collaboration, and self- fabrication increase the responsibility of everybody, which should be an excellent base for a sustainable consumer- producer-relationship.

Isanaka and Liou [37] summarized the roles of AM technologies to help establish a Cyber-Enabled Manufacturing environment by printing or embedding sensors and actuators in the proper locations. Such a networked environment is vital for sustainable quality control, and timely and predictive maintenance of the manufacturing equipment.

Brackett et al. [38] presented an overview of the issues and opportunities for the application of topology optimization methods for AM. The paper discussed analysis issues such as maximum geometric resolution for fine features, manufacturing constraints, and workflow modifications required to handle the geometric complexity in the post optimization stages. The manufacturing issues discussed included the potential for realizing intermediate density regions, in the case of the solid isotropic material with penalization (SIMP) approach, the use of small scale lattice structures, the use of multiple material AM processes, and an approach to including support structure requirements as a manufacturing constraint. Material and material-specific process development are key drivers toward realizing energy efficiency and reducing environmental footprint.

Morrow et al. [39] investigated three case studies to demonstrate the extent to which Direct Metal Deposition (DMD)-based manufacturing of molds and dies can currently achieve reduced environmental emissions and energy consumption relative to conventional manufacturing pathways. Laser-based remanufacturing of tooling also seems to reduce cost and environmental impact simultaneously, especially as the scale of the tool increases.

Huang et al. [40] presented the societal impact of additive manufacturing from a technical perspective. Their paper highlighted additive manufacturing in the following areas: customized healthcare products to improve population health and quality of life, reduced environmental impact for manufacturing sustainability, and simplified supply chain to increase efficiency and responsiveness in demand fulfillment. They highlighted the need for further research in the areas of life cycle energy consumption evaluation and potential occupation hazard assessment for additive manufacturing.

Bonnard et al. [41] proposed to use the STEP-NC concept, which contains high-level information such as simulation data, multi-material parts and inner structures, in order to integrate AM processes in a complete STEP-NC digital chain in accordance with ISO TC 184/SC 1 standards. The purpose of the proposed numerical chain was to define a global process control from knowledge of process obtained from experimentation, measurements, and simulations. Sustainability can potentially be a goal in such global process control.

## 4. Sustainability Characterization

More recently the sustainable benefits of AM have been widely hypothesized both by industry and academia. But to promote the widespread implementation of the technology, we need scientific data through established measurement methods [42].

There has been reported work on performance evaluation for AM processes (example: [43–45]). Appropriate benchmark parts have been designed for performance evaluation of AM systems and processes, and provide helpful decision support data. Several benchmark studies have been carried out to determine the levels of dimensional accuracy and surface quality achievable with current AM processes. Besides the process and the material, there may be other factors, such as the building style and specific process parameters that may affect the accuracy and finish of the part. Moving towards sustainable manufacturing, measurement science methods need to be developed and standardized for AM to assess and value the impacts of sustainability.

### 4.1 Sustainability Characterization Methodology

Today manufacturing industries are being forced to create and deliver quality products while decreasing their environmental impact [46]. Transforming the environmental practices of manufacturing companies from a human experience base to science-based practices can be achieved through science-based sustainability characterization [47]. This characterization will include information for various performance indicators, which will be crucial in determination of the sustainability of a unit manufacturing process (UMP). UMPs are the individual steps that transform raw material into a finished product by adding energy [48]. Selective Laser Sintering, Stereo Lithography, and Fused Disposition Modeling are examples of UMPs applicable to the category of AM.

**Fig. 2:**
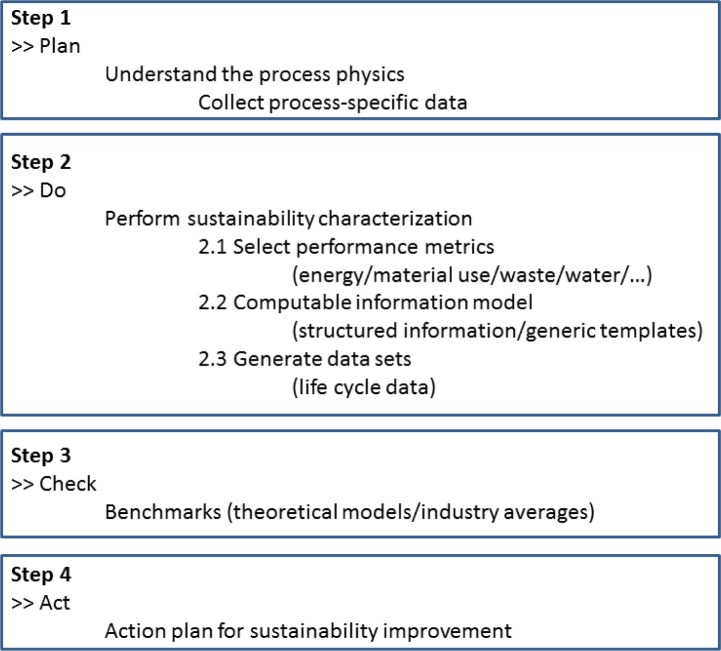
Sustainability characterization guide

Figure 2 presents an outline of a proposed sustainability characterization guide to provide a measurement framework for improving the sustainability of manufacturing processes and for consistently comparing different manufacturing processes for sustainability. The guide is yet to be formally developed and is presently an active Work Item within the ASTM E60.13 standards subcommittee on sustainability characterization for manufacturing processes [49]. In general, the proposed sustainability characterization guide includes four main steps. Step 1 involves understanding the process physics and collecting relevant data pertaining to the process. Step 2 includes performing the actual sustainability characterization [47]. Here the first part consists of defining the key performance indicators and their common computable metrics. Performance indicators can be broken down into two categories: input and output indicators. Examples of input indicators include water use, energy use, and material use. Examples of output indicators include product, solid waste, liquid waste, and air emissions [50]. The second part of the methodology involves determining analytics that can be used to calculate UMP sustainability, and incorporating the analytics within an information model. The third part involves applying manufacturing process-specific data sets to provide evidence in support of the information models and enable execution of computable metrics. This generates the life cycle inventory (LCI) data specific to the process. Step 3 of the sustainability characterization guide could include comparing the sustainability related data generated against other manufacturing processes or industry averages in general. Based on the results from Step 3, an action plan for improvement is developed in Step 4. The authors suggest that AM processes must be characterized for sustainability to truly understand and appreciate the environmental impact beyond just postulations and suggestions based on statically insignificant data.

When characterizing for sustainability, all aspects of the entire AM process from raw material preparation to pre-processing, actual fabrication, post-processing, to final part must be meticulously examined and appropriate measurement methods determined to account for sustainability. Comparisons to established processes, such as powder metallurgy which is considered sustainable since it can use recycled material [51], will need to be performed to better understand the sustainability aspects. A way to theoretically model sustainability for AM needs to be investigated. Prior research to theoretically model sustainability of other manufacturing processes based on energy use can be useful [52, 53].

As a disruptive technology there are potential benefits that could be realized with the use of AM in production of end-usable and critical parts. More importantly there is the potential for AM to boost the sustainability of the manufacturing industry in economic, environmental, and social terms. AM is particularly suited for industries in which mass customization, light weighting of parts, and shortening of the supply chain are economically valuable, particularly in fields such as the medical, dental, automobile, and aerospace industries [54].

## 5. Research Perspectives

There are still research gaps to be explored to understand and characterize sustainability of AM processes. Science-based sustainability characterizations for different AM technologies and processes need to be developed. Sustainability metrics and the corresponding measurement science need to be developed in conjunction with standards organizations and other standards development efforts. Besides these, the general challenges and research for AM to achieve a wider range of applications are still relevant and applicable to promote sustainability [55, 56]. These challenges include: process control with better feedback control systems and metrics to improve the precision and reliability of the manufacturing process; improved surface finishes of products; validation and demonstration for structural integrity of components; extensive testing, demonstration, and data collection for decision support; energy and material efficient AM systems; efficient processes to create metal powders and maintain shelf life; efficient post-processing; non-toxic and reusable materials; material and multi-material recycling and reuse strategies; integration of existing waste streams; alternative materials; carbon mapping tools; improved support structures generation strategies with less waste; design optimization and simulation tools for minimizing material and maximizing process efficiency; closed-loop remanufacturing solutions; development of distributed supply chain models; and models for reverse logistics and application-specific solutions.

## 6. Relevant ASTM Standards Organizations

ASTM International develops standards relevant to both sustainable manufacturing and AM.

### 6.1 ASTM Sustainable Manufacturing

To facilitate the development and use of sustainable manufacturing processes, ASTM International’s Committee E60 on Sustainability has created a new subcommittee, E60.13 on Sustainable Manufacturing [57, 58]. The subcommittee E60.13 is currently pursuing four standards’ work items namely: WK35702, New Guide for Evaluation of Environmental Aspects of Sustainability of Manufacturing Processes; WK35703, New Terminology for Standard Terminology for Sustainable Manufacturing; WK35705, New Guide for Sustainability Characterization of Manufacturing Processes; and WK38312, New Classification for Waste Generated at Manufacturing Facilities and Associated Claims. NIST is playing a leadership role in the E60.13 subcommittee efforts.

### 6.2 ASTM AM Technology

ASTM’s AM Technology standards are intended to promote knowledge of the industry, help stimulate research, and encourage the implementation of technology. The standards are developed by ASTM Committee F42 and define terminology, measure the performance of different production processes, ensure the quality of the end products, and specify procedures for the calibration of AM machines. The ASTM F42 technical subcommittees are working towards standards in materials and processes, terminology, design and data formats, and test method [59]. NIST actively leads some of the ASTM F42 subcommittee efforts.

More recently ISO and ASTM International signed an agreement to increase cooperation for co-developing International Standards for AM [60].

## 7. Conclusion

Additive manufacturing is a promising approach to build complex shapes, customized parts, or small batches of products where creating a mold or using a machining process would be wasteful and time consuming. More efforts are needed to accelerate the maturity of AM technology for critical components and mass production. The sustainability aspects of AM may offer advantages for industry to embrace the technology. But today there is a lack of measurement science to truly understand AM sustainability. Accordingly in this paper, we first presented related literature on the environmental impacts of AM. Next, we proposed an outline for a sustainability characterization guide to serve as a reference for the community to benchmark AM processes for sustainability. The guide is yet to be formally developed and is presently an active work item within the ASTM E60.13 committee. Finally we presented research perspectives along with a description of relevant ASTM standards organizations.

## Figures and Tables

**Table 1:** Key AM advantages relevant to sustainable manufacturing

Less waste because of the nature of the additive process, unlike parts that are stamped or sculpted out of a larger piece of material No specialized tooling or fixtures required for AM Ability to build functionally light weight parts, while maintaining strength Reduces the need for large amounts of raw material within the supply chain and transportation Largely material efficient when compared with traditional machining and casting Ability to produce optimized geometries with near-perfect (compared with wrought material) strength-to-weight ratios Less impact of the part over its life cycle, resulting in a lower carbon footprint, less embodied energy, and better economic model Ability to create on-demand spare parts, reducing or eliminating inventory

## Footnotes

1 Certain commercial equipment, instruments, or materials are identified in this paper to foster understanding. Such identification does not imply recommendation or endorsement by the National Institute of Standards and Technology, nor does it imply that the materials or equipment identified are necessarily the best available for the purpose.
